# Recent Advances in CRISPR/Cas Technologies for Biological Discovery, Therapeutics, and Diagnostics

**DOI:** 10.3390/biom16091268

**Published:** 2026-09-02

**Authors:** Moon-Soo Kim, Hae Sol Do, Hye Yeon Jang, Kang Eun Lee, Sun Ju Lee

**Affiliations:** Department of Innovative Drug Discovery and Development, College of Pharmacy, Keimyung University, 10, Daegu 42601, Republic of Korea; 5968637@stu.kmu.ac.kr (H.S.D.); 5902852@stu.kmu.ac.kr (H.Y.J.); 5968728@stu.kmu.ac.kr (K.E.L.); 5968745@stu.kmu.ac.kr (S.J.L.)

**Keywords:** CRISPR-Cas, genome editing, therapeutic target discovery, therapeutics, neurological disorder, infectious disease, diagnostics

## Abstract

The clustered regularly interspaced short palindromic repeats (CRISPR)/CRISPR-associated protein (Cas) system began as a tool for programmable genome editing. CRISPR/Cas technologies have evolved into a versatile platform for functional genomic screening, epigenome editing, therapeutic target discovery, and highly sensitive molecular diagnostics. New effectors and engineered variants continue to push these applications into personalized medicine and point-of-care testing. Here, this review highlights recent advances in therapeutic target discovery and therapeutic and molecular diagnostic development utilizing CRISPR/Cas technologies. We discuss how CRISPR interference (CRISPRi), CRISPR activation (CRISPRa), base editing, and prime editing have improved our understanding of disease mechanisms, while creating new opportunities for therapeutic intervention. Current applications in cancer immunotherapy, infectious disease, and neurological disorders are also discussed. In diagnostics, CRISPR-based platforms enable sensitive detection of infectious pathogens, cancer biomarkers, and genetic disorders using programmable nuclease activity in both laboratory and point-of-care settings. Collectively, these advances continue to expand the role of CRISPR/Cas technology across biological discovery, disease diagnostics, and therapeutic development, driving progress in precision medicine.

## 1. Introduction

The Clustered Regularly Interspaced Short Palindromic Repeats (CRISPR) are arrays of short, repeated DNA sequences separated by unique spacer sequences, which were first discovered in the *Escherichia coli* genome [1,2]. Later, evidence demonstrated that the CRISPR-Cas system functions as an adaptive immune system in archaea and bacteria [2,3]. This CRISPR system allows organisms to preserve foreign DNA fragments from viruses or plasmids, then use them as a genetic memory to recognize and destroy future invasive factors [2,3].

**Figure 1:**
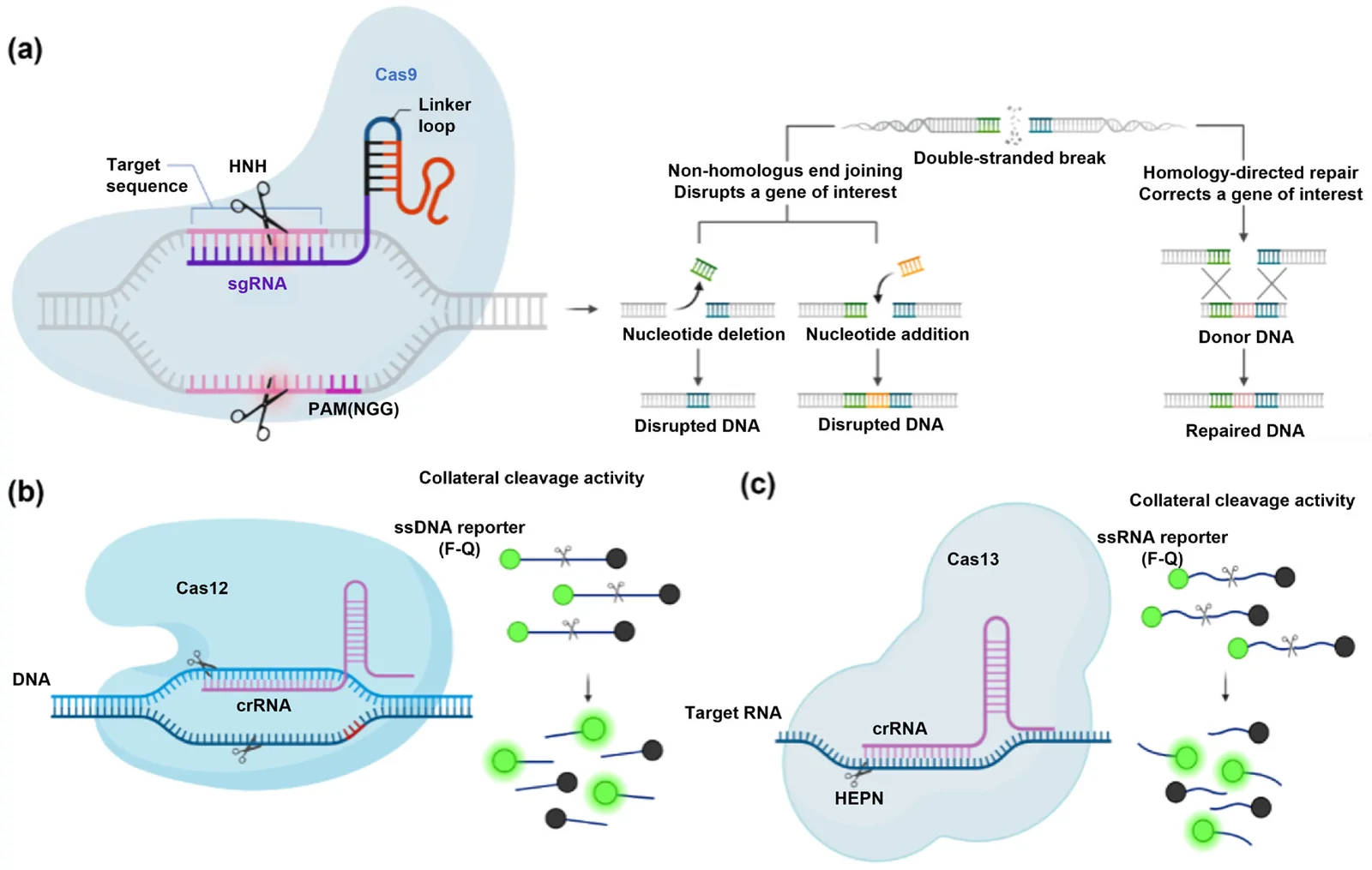
Overview of the molecular mechanisms of CRISPR-Cas systems. (**a**) Cas9 recognizes a protospacer adjacent motif (PAM)-containing target DNA via sgRNA and introduces a site-specific double-stranded break (DSB), which is repaired through non-homologous end joining (NHEJ) or homology-directed repair (HDR). (**b**) Cas12 recognizes target double-stranded DNA (dsDNA) through crRNA, performs sequence-specific cis-cleavage on the target DNA, and subsequently exhibits trans-cleavage activity toward nonspecific single-stranded DNA (ssDNA) reporters. (**c**) Cas13 targets single-stranded RNA (ssRNA) via crRNA and, upon activation, induces collateral cleavage of surrounding ssRNA reporters through its Higher Eukaryotes and Prokaryotes Nucleotide-binding (HEPN) nuclease domains, enabling sensitive RNA detection.

Genome editing changed dramatically with the arrival of the CRISPR/Cas system a few decades ago [4]. The CRISPR system functions through a ribonucleoprotein (RNP) complex composed of a Cas nuclease and a guide RNA (gRNA), which together mediate target DNA recognition and cleavage. The guide RNA typically consists of two RNA components—the CRISPR RNA (crRNA), which provides target sequence specificity, and the trans-activating CRISPR RNA (tracrRNA), which associates with the Cas protein and facilitates RNP assembly [4]. In many genome-editing applications, these two RNAs are fused into a single-guide RNA (sgRNA) that retains both targeting and structural functions [5,6]. The targeting region in the sgRNA contains about 20 nucleotides complementary to the target DNA sequence, which determines the precise target position for a Cas protein to cut. The CRISPR-Cas system comprises several Cas protein families, among which Cas9, Cas12, and Cas13 are the most extensively studied (Figure 1). CRISPR was subsequently demonstrated to be effective in mammalian cells, confirming its versatility, efficiency, and ease of implementation for genome editing [5,6]. Since then, it has become the most widely used genome-editing technology owing to its simplicity, high efficiency, and ease of reprogramming.

**Figure 2:**
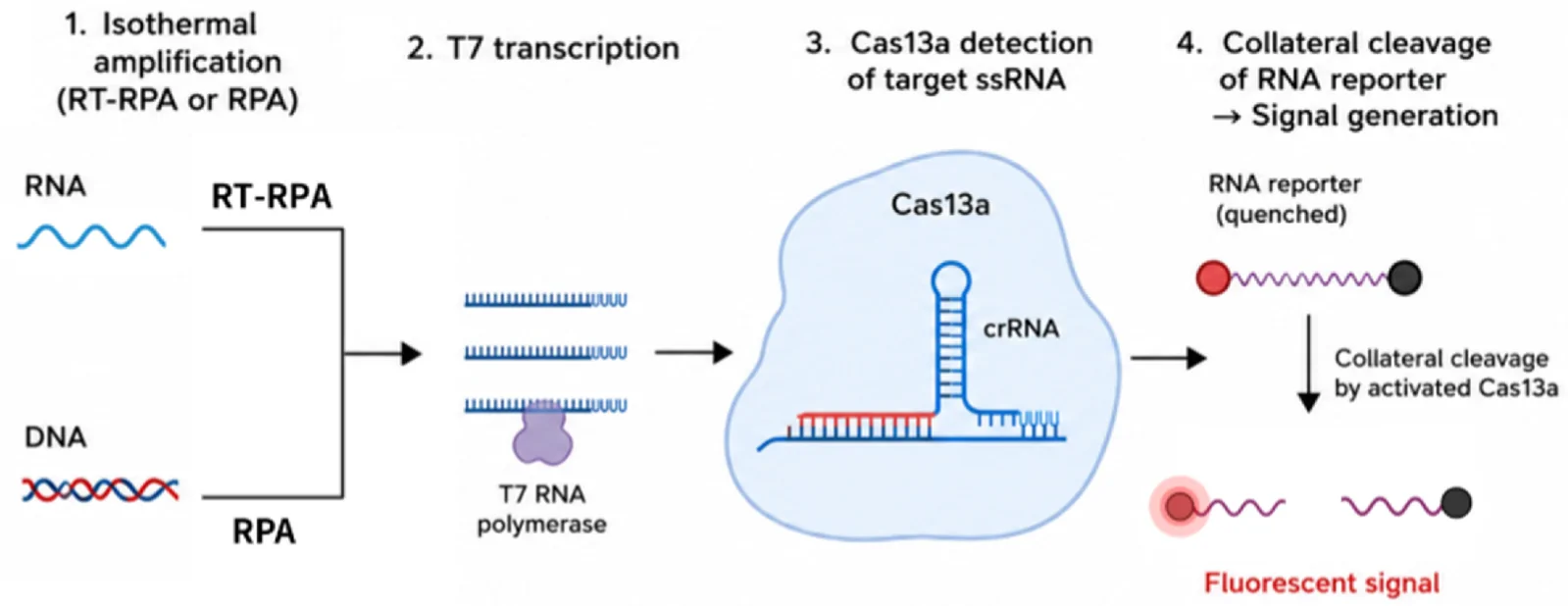
SHERLOCK (Specific High-sensitivity Enzymatic Reporter unLOCKing), a Cas13a-based RNA detection system. Target RNA or DNA is first amplified via isothermal amplification (reverse transcription recombinase polymerase amplification (RT-RPA) or recombinase polymerase amplification (RPA)) and converted into RNA transcripts by T7 RNA polymerase. Cas13a, guided by a crRNA complementary to the target ssRNA, is then activated upon target recognition, triggering collateral cleavage of quenched RNA reporters and generating a detectable fluorescent signal.

CRISPR was first discovered in 1987, but it took nearly two decades before researchers recognized its role as a bacterial adaptive immune system, established between 2005 and 2007 [2]. In 2012, it was successfully repurposed as a programmable genome-editing tool, revolutionizing the field of genetic engineering [4]. Following its establishment as a genome-editing platform, CRISPR technology rapidly diversified into a wide range of applications. Base editors were among the first extensions, allowing precise single-nucleotide changes without cutting both DNA strands. Subsequently, the CRISPR-based diagnostic platform SHERLOCK (Specific High-sensitivity Enzymatic Reporter unLOCKing) turned Cas13a’s collateral cleavage activity into an ultrasensitive nucleic acid detection tool (Figure 2) [7]. Paired with isothermal amplification, it can pick up both RNA and DNA targets—a capability already put to use in diagnosing Zika and dengue virus infections. Around the same time, DETECTR (DNA Endonuclease-Targeted CRISPR Trans Reporter) took a parallel approach, exploiting Cas12a’s collateral single-stranded DNase activity instead [8]. Its first real-world test came in human papillomavirus (HPV) diagnosis, where it detected target DNA quickly and with high sensitivity—an early sign of what CRISPR-based diagnostics could offer at the point of care [8]. Another major advancement is the RNA Editing for Programmable A-to-I Replacement (REPAIR) system, which took a different route: it pairs catalytically inactive Cas13 with the ADAR (adenosine deaminase acting on RNA) enzyme to convert adenosine to inosine at programmable sites in RNA transcripts [9]. Because cells read inosine as guanosine during translation, this swap corrects the RNA sequence transiently, leaving the genome untouched [9].

CRISPR-based technologies have come a long way, but several obstacles still stand between the lab and the clinic. Off-target effects, inconsistent editing outcomes, and cell-type-dependent variability can all undercut editing efficiency and specificity [10,11,12]. In addition, the safe and efficient in vivo delivery of CRISPR components remains a major obstacle, as current viral vectors and nonviral nanocarriers still face limitations related to targeting specificity, delivery efficiency, immunogenicity, and payload capacity [13,14]. Clinical trials have already reached several genetic disorders and cancers, but neurological disease, infectious disease, and tissue regeneration are only just starting to be explored [15,16,17]. Closing these gaps will likely take sharper editing precision, better Cas variants and delivery vehicles, and platforms robust enough for everyday clinical use.

Once purely a genome-editing tool, CRISPR is now reshaping several corners of biomedicine at once. Here, we walk through the molecular mechanisms behind CRISPR/Cas systems and look at where they’re being put to use—genome editing, therapeutic target discovery, therapeutics and molecular diagnostics—before turning to the obstacles still standing between the lab and the clinic. What emerges is a technology still finding new applications faster than the field can fully absorb them.

## 2. Cas Proteins and DNA-Repair Mechanisms

### 2.1. Comparative Features of Cas Proteins

The protospacer adjacent motif (PAM) is a short DNA sequence, typically 2–6 bp in length, located immediately adjacent to the target site [3]. PAM recognition serves as a prerequisite for CRISPR-Cas activity by enabling the Cas protein to identify target DNA, initiate local DNA unwinding, and subsequently assess guide RNA complementarity [4,18]. Because PAM recognition is Cas protein-specific, different Cas nucleases require distinct PAM sequences for target binding and cleavage (Table 1) [3]. In bacterial genomes, the absence of PAM sequences adjacent to the CRISPR spacer-derived target sites prevents self-recognition and cleavage of the host genome, thereby ensuring discrimination between self and foreign DNA [2]. Cas9 is the most widely used nuclease and functions as an RNA-guided endonuclease that introduces site-specific double-stranded DNA (dsDNA) breaks. It typically recognizes a guanine-rich PAM sequence (5′-NGG-3′) and generates blunt-ended DNA cleavage, making it well suited to applications that demand high efficiency, such as gene knockout and high-throughput screening. However, gRNA design and PAM constraints must be carefully managed for specificity [4]. In contrast, Cas12 recognizes thymine-rich PAM sequences, such as 5′-TTTV-3′, and produces staggered DNA breaks. Upon target recognition, activated Cas12 also exhibits collateral cleavage activity against non-target single-stranded DNA (ssDNA), a property that has been widely exploited for nucleic acid diagnostics [19]. Unlike Cas9 and Cas12, Cas13 specifically targets single-stranded RNA (ssRNA) rather than DNA and therefore does not directly modify the genome. This RNA-targeting capability enables transient gene regulation and antiviral applications, particularly against RNA viruses, without introducing permanent genomic alterations [7]. Furthermore, Cas12 and Cas13 exhibit collateral cleavage activity, whereby activation upon target recognition triggers indiscriminate cleavage of nearby single-stranded nucleic acids. This unique property has been widely exploited in nucleic acid diagnostic platforms [7,8], whereas Cas9 is primarily employed for precise genome editing because it lacks collateral nuclease activity. Cas9 is widely used for precise genome editing because, unlike Cas12 and Cas13, it does not exhibit robust collateral nuclease activity. That said, a recent study has found that activated Cas9 can show limited nonspecific ssDNA trans-cleavage under certain conditions—though this activity remains far weaker and more restricted than the collateral cleavage seen with Cas12 or Cas13 [20].

Cas12 (Cpf1) recognizes a T-rich PAM and cleaves distal to it, producing sticky ends and extending editing into AT-rich regions that Cas9 struggles to reach; it also processes its own crRNA, simplifying multiplex targeting [21]. Ultracompact nucleases such as Cas12f1, which are substantially smaller than conventional Cas9 and Cas12a proteins, can alleviate the packaging constraints of adeno-associated virus (AAV) vectors and facilitate single-vector delivery. An engineered Cas12f1 system delivered via a single AAV vector in a mouse model of retinitis pigmentosa achieved therapeutically relevant editing and partially restored retinal structure and visual function [22]. This compact architecture provides an alternative to more complex delivery strategies, such as dual-AAV systems or intein-mediated protein trans-splicing, that are often required for larger CRISPR components [23]. However, these therapeutic results were obtained in a mouse model following local subretinal administration [22]. Further studies are therefore needed to determine whether ultracompact Cas systems can achieve comparable efficacy and safety with systemic delivery and in tissues beyond the eye.

Cas13 targets RNA rather than DNA, enabling transient modulation of gene expression without permanently altering the genome [21]—an advantage for diseases where permanent editing would be risky, such as those requiring careful control of gene dosage, or for antiviral therapies. Cas13 has been explored against RNA viruses including severe acute respiratory syndrome coronavirus 2 (SARS-CoV-2), HIV, dengue, and influenza, since guide sequences can be swapped quickly to keep pace with emerging variants [24], unlike traditional antivirals and vaccines built around fixed targets. Key obstacles remain, however: collateral cleavage causes nonspecific RNA degradation, delivery is inefficient, immunogenicity is a concern, and no clear ethical framework yet exists for clinical translation [24]. Addressing these will require better crRNA design, improved delivery vehicles, and rigorous preclinical testing [24]. Notably, the same collateral cleavage that limits Cas13′s therapeutic use is what makes it valuable for diagnostics, since the sensitivity from degrading non-target RNA is exactly what diagnostic platforms need.

In short, Cas9 is well suited to applications requiring efficient and permanent genome modification; ultracompact Cas12 orthologs—distinct from larger Cas12a variants such as AsCas12a or LbCas12a, which approach SpCas9 in size—offer particular advantages for in vivo applications constrained by delivery capacity; and Cas13 enables transient RNA targeting, making it attractive for reversible gene regulation, antiviral applications, and molecular diagnostics. However, these platforms differ substantially in their translational maturity, with Cas9 having the most extensive clinical experience, whereas ultracompact Cas12 and Cas13 systems remain largely at earlier stages of therapeutic development. Platform selection therefore depends on the desired duration and type of editing, delivery constraints, target accessibility, and acceptable safety profile.

### 2.2. DNA-Repair Mechanisms

Cas9-induced cleavage of the target DNA generates a double-strand break (DSB) that must be repaired by the cell’s endogenous DNA repair machinery to restore genome integrity. Following Cas9-mediated cleavage, the broken DNA ends can be rejoined through end-joining repair pathways, resulting in small insertions or deletions (indels) at the cleavage site. In contrast to homology-directed repair (HDR), which uses a homologous DNA template to introduce defined sequence changes, end-joining repair does not require an exogenous donor template. Consequently, processing of broken DNA ends before ligation can alter the original sequence, frequently resulting in insertions or deletions (indels) at CRISPR-Cas9-induced double-strand breaks [25].

At the molecular level, the formation of an indel depends on how the two DNA ends generated by the Cas9-induced DSB are processed and rejoined. If the broken ends are not directly compatible for ligation, nucleotide removal or addition can occur during end processing before the ends are re-ligated. Nucleotide removal can generate deletions, whereas nucleotide addition or fill-in synthesis can generate insertions. In addition, sequence homology between DNA ends can influence the repair outcome. Large-scale analysis of more than 1000 genomic target sites demonstrated that CRISPR-Cas9-induced indel formation is not completely random but is influenced by local sequence context. Deletion patterns were frequently associated with microhomology at the repair junctions, whereas recurrent insertions often contained nucleotides matching sequences adjacent to the cleavage site. These findings indicate that local DNA sequence features influence DSB repair outcomes and contribute to the reproducible, target-specific patterns of indel formation [26].

The resulting indels can subsequently alter the coding sequence when the Cas9 cleavage site is located within a protein-coding region. If an insertion or deletion alters the coding sequence by a number of nucleotides that is not a multiple of three, the downstream reading frame is shifted, resulting in a frameshift mutation. This changes the amino acid sequence downstream of the editing site and can result in the introduction of a premature termination codon, potentially producing a truncated or non-functional protein. Consequently, frameshift-inducing indels can lead to loss of gene function and are commonly exploited to generate CRISPR-Cas9-mediated gene knockouts. However, not every indel produces a frameshift: insertions or deletions of three or multiples of three nucleotides can preserve the reading frame and may therefore retain partial or substantial protein function. Thus, the functional outcome of CRISPR-Cas9 editing depends on the precise size and position of the indel generated at the target locus [25,26]. Overall, CRISPR-Cas9-mediated gene knockout can therefore be understood as a sequential process in which Cas9 generates a sequence-specific DSB, the resulting DNA ends undergo end-joining repair and end processing, and nucleotide loss or addition produces an insertion or deletion at the repair junction. When such an indel disrupts the coding frame, a frameshift can occur, potentially leading to premature termination and loss of protein function. Importantly, the precise repair outcome is influenced by the sequence context of the target site, and large-scale analyses have demonstrated that some loci exhibit highly reproducible indel profiles whereas others produce a broader range of repair products [26].

## 3. Gene and Genome Editing

### 3.1. CRISPR-Mediated KO and KI Mechanisms

Cas9-induced DSBs are primarily repaired by NHEJ, which rejoins broken DNA ends without a donor template and often generates small indels, leading to gene knockout (KO). Alternatively, HDR uses a donor DNA template to enable precise gene correction or knock-in (KI), although it requires DNA end resection and is generally less efficient [12,27]. Because both pathways act on the same DSB, they are not independent: DNA-dependent protein kinase (DNA-PK) inhibition increased HDR-mediated KI and reduced NHEJ-associated indels, but this was accompanied by an increase in microhomology-associated deletions attributable to alternative end joining (alt-EJ) [27]. This indicates that suppressing NHEJ does not simply convert breaks to HDR outcomes; rather, it redistributes repair across competing pathways, so the resulting balance of indel- versus template-directed edits is not fully predictable from cleavage efficiency alone.

This competition is further shaped by cell-cycle status: NHEJ can occur throughout the cycle, whereas HDR is favored in S/G2M phases when a sister chromatid is available as a template [28]. Small molecules that enrich cells in the S/G2/M phases promote HDR over NHEJ, thereby enhancing CRISPR/Cas9-mediated knock-in (KI) efficiency through increased CtIP (C-terminal binding protein-interacting protein)-mediated DNA end resection and replication protein A (RPA) loading [28]. However, the magnitude of this HDR-promoting effect varied by cell type [28]. Together, these findings indicate that high Cas9 cleavage efficiency alone does not guarantee high precise-editing efficiency, since the outcome depends on ongoing competition among NHEJ, HDR, and alternative repair pathways, modulated by cell-cycle and cellular context.

HDR is essential for precise KI genome editing, but in cells it competes with NHEJ, resulting in low efficiency. In particular, NHEJ predominates during the G1 phase, restricting HDR-based KI and leading to heterogeneous editing outcomes within the same cell population. To overcome this limitation, recent studies have explored pharmacological modulation of DNA repair pathways to promote HDR over NHEJ and improve genome-editing precision [29,30,31]. For example, the CRISPY mix, a combination of small molecules, significantly enhanced HDR-mediated targeted nucleotide substitution and KI efficiency in human induced pluripotent stem cells (iPSCs) [29]. The CRISPY mix markedly improved KI efficiency, achieving up to a sevenfold increase in targeted insertion [29]. Fusion of Cas9 with a HUH endonuclease to tether single-stranded oligodeoxynucleotide (ssODN) donors has been shown to markedly enhance HDR, achieving up to 30-fold increases in efficiency without elevating off-target activity [30]. In vivo validation further supports the utility of HDR enhancers; treatment of bovine embryos with the RAD51 activator RS-1 nearly doubled knock-in rates, while maintaining normal development [31]. These observations underscore that modulation of DNA repair pathways—through suppression of NHEJ or activation of HDR factors—can bias repair outcomes toward HDR, thereby improving both knock-in efficiency and editing precision [29,30,31].

Cas12a provides an alternative platform for DNA-based gene KO and KI because it recognizes T-rich PAM sequences and generates staggered DNA double-strand breaks, thereby facilitating precise genome editing [32]. Cas12a can tolerate certain crRNA–DNA mismatches, resulting in unintended cleavage at off-target sites. Structure-guided engineering of Cas12a variants has reduced this off-target activity while maintaining efficient on-target editing [32]. Cas13 is not primarily used for permanent genomic KO or KI unlike Cas9. Instead, its RNA-targeting activity enables transient transcript manipulation without introducing a genomic DSB. This feature can be useful when permanent DNA modification is undesirable, although Cas13-based systems have their own specificity concerns, including unintended RNA cleavage and off-target transcript editing. These limitations can be addressed by engineering Cas13 or its associated RNA-editing components, as demonstrated by the development of more specific RNA-editing systems [9].

### 3.2. Base Editor (BE) and Prime Editing (PE)

CRISPR/Cas9-based gene correction typically relies on double-stranded DNA breaks and homology-directed repair (HDR), which is often inefficient and prone to unintended indels. Base Editor 3 (BE3) addresses this by fusing a cytidine deaminase to Cas9 nickase along with a uracil DNA glycosylase inhibitor (UGI). The cytidine deaminase converts cytosine (C) to uracil (U), creating a U–G mismatch that is subsequently resolved by cellular DNA replication or repair to produce the desired C-to-T (or G-to-A) substitution without generating a double-strand break [33]. In this system, no HDR is required, and indel formation is minimal. Reported editing efficiencies reach up to 37%, and the system has corrected disease-associated mutations in genes such as APOE4 and TP53 [33].

Prime editing goes beyond base editing: it allows targeted insertions, deletions, and all 12 possible base-to-base conversions, and it does so without double-strand breaks or a donor DNA template [34]. The system pairs a catalytically impaired Cas9 nickase with an engineered reverse transcriptase, while a prime editing guide RNA (pegRNA) directs the complex to its target and encodes the intended edit. Once Cas9 nicks the DNA, the exposed strand primes reverse transcription, and the edited sequence is synthesized directly at the target site. The optimized PE3 (Prime Editing 3) system edits with roughly 20–50% efficiency and generates far fewer indels than HDR-based methods. Further, prime editing has corrected mutations underlying sickle cell disease and Tay–Sachs disease as well as introduced the naturally occurring protective G127V variant in the prion protein gene (*PRNP*), which confers resistance to prion disease, into human cells [34]. Most point mutations and small indels can now be corrected without HDR, which makes prime editing one of the more versatile tools in the CRISPR toolbox [34]. In a more recent study [35], a prime editor protein (PE7) was fused to the RNA-binding domain La as a key determinant of prime editing efficiency. This system enhanced prime editing with expressed pegRNAs and engineered pegRNAs optimized for La binding, achieving editing efficiency across disease-relevant targets while maintaining low off-target activity [35].

Taken together, BE and PE exhibit distinct strengths and limitations in terms of delivery, editing scope, and off-target activity. BE, though generally smaller in cargo size than PE, still frequently exceeds single-AAV packaging capacity once the deaminase domain—and, for cytosine base editors specifically, one or two copies of UGI—is added to Cas9, often necessitating dual-AAV split systems or compact Cas9 orthologs for in vivo delivery [36,37]. BE also has more off-target activity than PE, for two main reasons. First, the deaminase domain can act stochastically on genomic DNA and RNA independent of Cas9 targeting, producing guide-RNA-independent edits genome-wide and transcriptome-wide; BE also retains the conventional Cas9-associated risk of editing at sequences with partial homology to the guide RNA. Second, PE’s multi-step mechanism—requiring successful primer binding site annealing, reverse transcriptase extension, and 3′ flap resolution—imposes sequential specificity checkpoints largely absent from BE, further constraining off-target editing. In contrast, PE relies on a multi-step recognition process involving protospacer binding by the Cas9 nickase, pegRNA primer-binding-site hybridization and reverse transcription at the nicked strand, and subsequent 3′-flap resolution. These additional sequence-dependent steps provide specificity checkpoints beyond Cas9–protospacer recognition, contributing to the generally low off-target activity reported for PE, particularly compared with conventional Cas9 nuclease editing [36]. BE may be preferable when the desired nucleotide conversion falls within its editing scope and high editing efficiency is required, whereas PE offers greater versatility by enabling all 12 base substitutions as well as targeted insertions and deletions. This broader editing scope, however, comes with greater delivery complexity and, in many settings, lower or more variable editing efficiency [36,37].

### 3.3. Cas13-Based Programmable RNA Editing

Most genome-editing tools permanently alter DNA, but REPAIR (RNA Editing for Programmable A-to-I Replacement) works differently: it targets RNA, pairing catalytically inactive Cas13b (dCas13b) with the adenosine deaminase acting on RNA 2 (ADAR2) deaminase domain to convert adenosine (A) to inosine (I) at guide RNA-specified sites in a transcript [9]. With this approach, mutations in AVPR2 (linked to X-linked nephrogenic diabetes insipidus) and FANCC (linked to Fanconi anemia) were corrected at the RNA level, leaving the genome untouched. The editing efficiencies ranged from 20–40%, reaching as high as 51% at selected target sites. A later version, REPAIRv2, used an engineered ADAR2 deaminase to sharpen specificity dramatically—transcriptome-wide off-target events dropped from 18,385 to just 20, with on-target editing largely unaffected [9]. That reversibility is REPAIR’s real advantage over DNA base and prime editing, which alter the genome permanently.

Beyond programmable RNA base editing, Cas13 also supports transcript knockdown and nucleic acid detection through the same crRNA-guided targeting mechanism, making RNA editing one application of a broader CRISPR platform [38]. A major challenge for therapeutic RNA editing is the large size of Cas13-ADAR editors, which generally exceeds the packaging capacity of adeno-associated virus (AAV) vectors [38]. To overcome this limitation, sequence mining identified the ultra-compact Cas13bt subfamily (775–804 amino acids), which retained efficient transcript knockdown activity in both bacterial and mammalian cells [38]. Fusion of catalytically inactive Cas13bt with ADAR2 generated compact RNA editors that fit within a single AAV vector while maintaining the ability to edit endogenous transcripts [38]. Although this strategy improved the feasibility of in vivo delivery, editing efficiency following AAV delivery remained lower than that achieved by plasmid transfection. In addition, off-target RNA editing and collateral RNase activity remain important concerns, requiring further optimization of deaminase specificity and the use of catalytically inactive Cas13 variants. Together, these findings indicate that efficient delivery and high RNA-editing specificity, rather than DNA damage, are the principal challenges for therapeutic Cas13-based RNA editing.

CRISPR-based therapies have advanced on several fronts, from higher editing efficiency to finer control over gene expression and disease-causing mutations. Among these advances, Three Prime Repair Exonuclease 1 (TREX1)—a major 3′→5′ DNA exonuclease—was identified as a key bottleneck limiting homology-directed repair (HDR)-mediated genome editing [10]. TREX1 degrades single-stranded donor DNA templates, thereby reducing HDR efficiency. Whether TREX1 is depleted genetically, inhibited pharmacologically, or simply outcompeted by chemically protected donor templates, the result is the same: more precise editing in both immortalized and primary cells. This makes TREX1 a promising biomarker for HDR efficiency—and a plausible target for improving CRISPR-mediated editing more broadly [10].

### 3.4. Off-Target Effects and Specificity in CRISPR Editing

One of the main challenges in CRISPR gene editing is off-target cleavage, in which nucleases cut DNA at sites other than the intended target. These unintended events can introduce mutations or chromosomal alterations and remain an important consideration for therapeutic applications. Several genome-wide methods have been developed to detect off-target cleavage. GUIDE-seq uses the integration of short double-stranded oligodeoxynucleotides at nuclease-induced DNA breaks to map cleavage sites across the genome [39], whereas DISCOVER-seq detects the recruitment of the DNA repair factor MRE11 to double-strand breaks, enabling unbiased identification of nuclease-induced cleavage sites in cells and in vivo [40]. In parallel, high-fidelity Cas9 variants, including SpCas9-HF1 [41] and HiFi Cas9 [42], have been engineered to reduce off-target activity by modifying Cas9–DNA interactions. However, increased fidelity can be accompanied by reduced editing activity at some target sites [41,42], highlighting the need to balance specificity and efficiency. Thus, the choice of off-target detection method and nuclease variant should be tailored to the experimental context and intended application.

## 4. Epigenome Editing

Beyond cutting DNA, CRISPR-Cas systems can also edit the epigenome—modifying DNA methylation and histone marks such as methylation and acetylation without ever breaking the double helix [43]. Many CRISPR-based transcriptional regulators such as catalytically inactive Cas9 (dCas9)-VP64 and dCas9-Krüppel-associated box (KRAB) function by recruiting epigenetic modifiers that activate or repress gene expression through targeted chromatin remodeling [44]. Chromatin accessibility itself is governed largely by histone acetylation, histone methylation, and DNA methylation. Acetylation tends to open chromatin, while deacetylation compacts it. Methylation is less predictable—its effect depends on which residue is modified and how many methyl groups are added (mono-, di-, or tri-methylation) [45]. Ultimately, these accessibility changes determine whether transcriptional machinery can reach the DNA at all [46]. Fusing the catalytic core of p300 (a histone acetyltransferase) to dCas9 enables targeted deposition of H3K27 acetylation (H3K27ac), thereby promoting gene expression [45,46].

SunTag variants of dCas9-VP64 build on the same idea, but add a signal amplification system that recruits multiple copies of transcriptional activators to a single locus [45]. This drives an active chromatin state marked by H3K27ac and H3K4 methylation, and transcription increases accordingly [45]. The synergistic activation mediator (SAM) system takes this further: engineered sgRNA scaffolds carrying MS2 aptamers recruit three activation domains—VP64, p65, and heat shock factor 1 (HSF1)—alongside dCas9. This design produces robust activation of endogenous genes and has enabled genome-scale gain-of-function screens [47]. Conversely, dCas9-KRAB recruits histone methyltransferases and histone deacetylases to establish repressive chromatin and silence transcription. Lysine-specific demethylase 1 (LSD1) achieves the same outcome through a different route: tethered to dCas9, it removes H3K4 methylation [45,48]. With so many tunable parts available, epigenome editing may be the most flexible approach yet to programmable gene regulation.

## 5. Gene Regulation Through CRISPRi and CRISPRa

**Figure 3:**
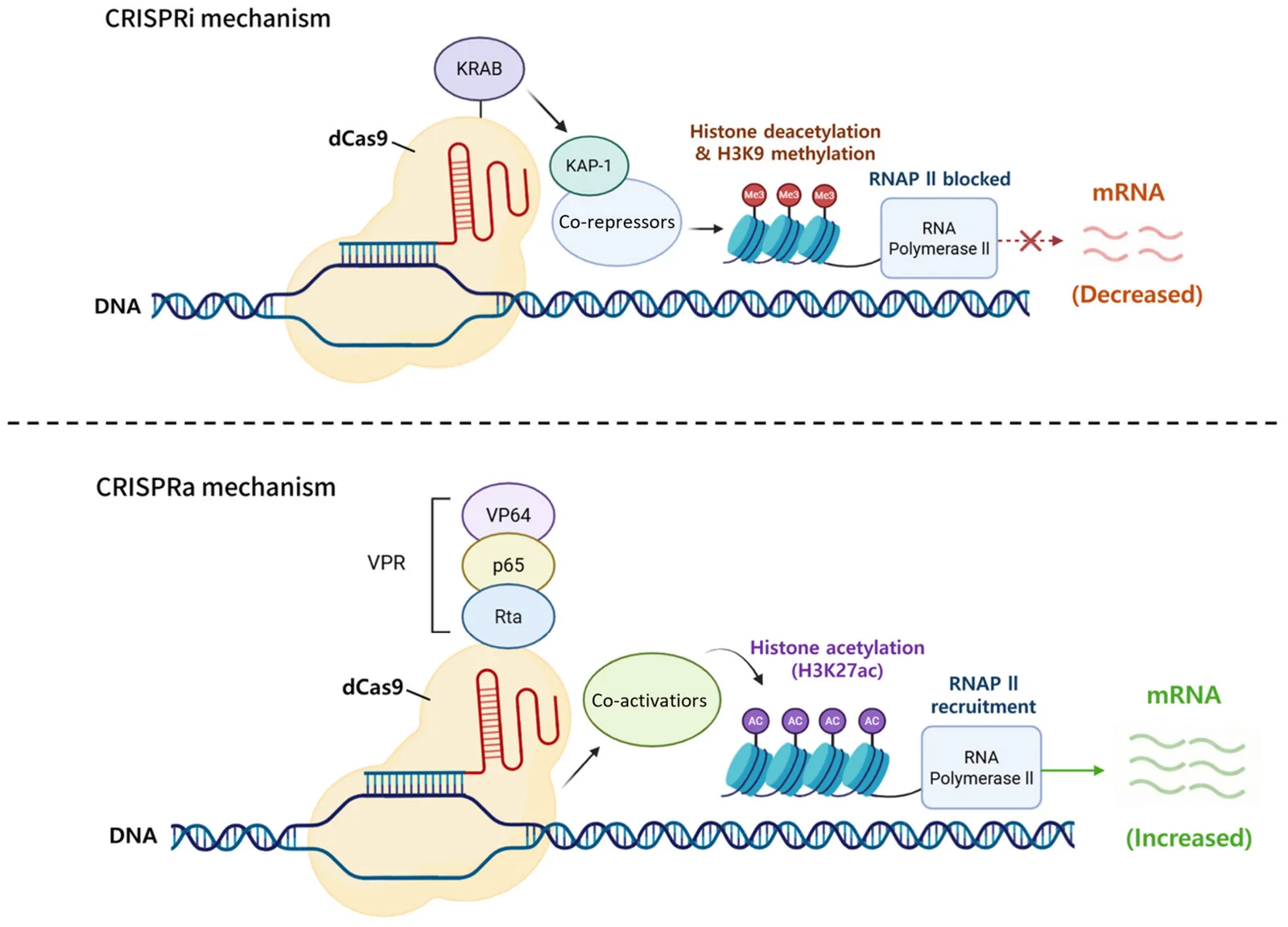
Mechanisms of CRISPR interference (CRISPRi) and CRISPR activation (CRISPRa). In CRISPRi (top), catalytically dead Cas9 (dCas9) is fused to the KRAB repressor domain, which recruits the KAP-1 co-repressor complex to induce histone deacetylation and H3K9 methylation, thereby blocking RNA Polymerase II and silencing target gene transcription. In CRISPRa (bottom), dCas9 is fused to the VPR transactivation complex (VP64-p65-Rta), which recruits co-activators that promote histone acetylation (H3K27ac) and RNA Polymerase II recruitment, resulting in increased mRNA expression.

CRISPR interference (CRISPRi) is based on a catalytically inactive Cas9 (dCas9) fused to transcriptional repressors such as KRAB. By binding to the transcription start site, this complex suppresses gene expression without inducing DNA cleavage, allowing highly specific transcriptional knockdown [49] (Figure 3). In contrast, CRISPR activation (CRISPRa) employs transcriptional activators such as the VPR complex (VP64-p65-Rta) to robustly enhance gene expression, with reported increases of 10- to 18-fold in neuronal differentiation markers in human induced pluripotent stem cells (iPSCs) (Figure 3) [50].

In practice, robust CRISPRi silencing in complex eukaryotic systems typically requires fusing dCas9 to a transcriptional repressor domain such as KRAB. A catalytically dead variant of the Cas12a (Cpf1) nuclease, which recognizes T-rich PAM sequences and requires only a crRNA, offers a complementary platform better suited to AT-rich target regions and can likewise be tethered to KRAB [43]. KRAB, a 75-amino acid domain found in eukaryotic zinc finger proteins, silences transcription by binding the KRAB-associated protein 1 (KAP-1) corepressor through a helix-helix interaction. KAP-1, in turn, assembles a chromatin regulatory complex whose repressive activity is thought to involve histone deacetylation, although the precise mechanism remains incompletely understood [51,52]. A related strategy fuses dCas9 to MAX-interacting proteins, which achieve comparable repression by recruiting the histone deacetylase Sin3 homolog [53,54].

CRISPRa strategies have similarly diversified. One approach bypasses conventional transcriptional activation domains by fusing dCas9 directly to the catalytic core of the histone acetyltransferase p300 [55]. This dCas9–p300 fusion catalyzes histone H3 lysine 27 acetylation (H3K27ac) at target enhancers—for example, the Fos enhancer—thereby promoting transcription of the associated gene [55]. Signal amplification can be achieved using the SunTag system, which consists of tandem peptide repeats that recruit multiple copies of a transcriptional activator to a single dCas9 molecule, resulting in gene expression levels up to 25-fold higher than those achieved with dCas9-VP64 alone [56]. Building on this scaffold, the dCas9-SunTag-P65-HSF1 (SPH) complex, which pairs multiple copies of the nuclear factor kB activator P65 with heat shock factor 1, is notably effective in heterochromatic regions that are otherwise difficult to activate [56]. The VPR (VP64-p65-Rta) system, which combines three transcriptional activation domains in tandem, induces stronger gene activation than VP64 or p65 alone and can be further enhanced using SunTag arrays [56]. More recently, fusion of dCas9-VPR with phase separation-prone proteins, such as nucleoporin 98 (NUP98) or fused in sarcoma (FUS), whose intrinsically disordered regions form multivalent condensates, has been shown to further enhance transcriptional activation by concentrating dCas9 complexes at target loci [57,58].

## 6. Therapeutic Target Discovery

CRISPR-based functional screening enables systematic identification of therapeutic targets involved in cancer development and immune regulation through both in vitro and in vivo studies. For example, CRISPR screening identified LIG1 as a therapeutic target whose inhibition sensitizes cancer cells to poly(ADP-ribose) polymerase (PARP) inhibitors, suggesting new opportunities for combination therapy and patient stratification [53]. In pancreatic cancer, an in vivo CRISPR screen identified the deubiquitinating enzyme USP15 (ubiquitin-specific peptidase 15) and the transcription-associated RNA processing factor SCAF1 (SR-related CTD-associated factor 1) as previously unrecognized tumor suppressors [59]. Loss of either USP15 or SCAF1 increased the sensitivity of pancreatic cancer cells to the PARP inhibitor olaparib and the chemotherapeutic agent gemcitabine, suggesting impaired DNA damage repair capacity. Analysis of patient samples further showed that approximately 37% of pancreatic ductal adenocarcinoma (PDAC) cases harbored deletions in either USP15 or SCAF1, suggesting that these alterations may identify a subset of patients who could benefit from targeted therapeutic strategies [59].

CRISPR-based functional screening has also accelerated the discovery of immunotherapeutic targets in colorectal cancer (CRC) [60]. An immune-focused CRISPR screen identified MBTPS1, a serine protease of the proprotein convertase subtilisin/kexin family, as a key regulator of antitumor immunity and a potential immunotherapy target [60]. The customized in vivo CRISPR-Cas9 screen was performed to identify genes encoding membrane and secreted proteins in CRC mouse models with different immune characteristics. MBTPS1 deficiency increased infiltration of CD8^+^ and CXCR3^+^CD8^+^ T cells, suppressed tumor growth, and enhanced the efficacy of anti-programmed cell death protein 1 (PD-1) therapy, resulting in complete tumor regression in preclinical models [60]. These findings suggest that MBTPS1 may serve as both a therapeutic target and a prognostic biomarker in colorectal cancer [60].

In leukemia, a Tudor domain-focused CRISPR screen targeting 59 Tudor domains identified SGF29 (SAGA-associated factor 29), a chromatin reader that recognizes trimethylated histone H3 lysine 4 (H3K4me3), as an essential regulator of leukemia maintenance [61]. Genetic depletion of SGF29 markedly suppressed leukemia progression both in vitro and in vivo. The CRISPR-Scan Assisted Drug Discovery (CRISPR-SADD) platform identified Cpd_DC60, a lead inhibitor targeting the SGF29 Tudor domain, which exhibited selective efficacy against leukemia while showing limited activity against solid tumors [61]. CRISPR-SADD linked therapeutic target discovery with drug development by enabling the identification of druggable protein surfaces for target-specific inhibitor design [61].

## 7. Therapeutic Development

### 7.1. Cancer Therapy

Cancer continues to be a significant worldwide health burden, and standard treatments are frequently restricted by recurrence and resistance. In this context, CRISPR-Cas technology has risen as a powerful tool to enable precise gene editing for new therapeutic approaches [12,62]. Recent studies have applied CRISPR/Cas technology either to reprogram immune cells for enhanced anti-tumor activity or to directly target tumors [11,15,16,63]. Gene editing in gastric and cervical cancer models, as well as clinical trials with PD1-edited T cells, demonstrate its clinical applicability [15,16,64,65]. Current findings highlight the therapeutic potential of CRISPR/Cas in cancer treatment, but several challenges remain. In particular, more efficient tumor-targeted delivery, reduced off-target editing, and further insight into tumor–immune interactions will be essential for broader clinical application [12,62,66].

At present, CRISPR/Cas is being explored in cancer therapy through two main approaches [11]. The first strategy involves ex vivo engineering of chimeric antigen receptor T (CAR-T) cells (patient-derived T lymphocytes engineered to recognize and kill tumor cells) to improve their anti-tumor activity and persistence. This is achieved by disrupting immune checkpoint molecules such as PD-1, a receptor on T cells that suppresses immune activation upon ligand binding, or by introducing cytokine genes [11]. Another strategy focuses on reprogramming the tumor microenvironment in vivo to overcome immunosuppression [11,63]. For example, Zhao et al. used bacterial-derived nanovesicles (NVs) to simultaneously deliver Cas9, sgRNA, and CpG DNA, inducing the conversion of tumor-associated macrophages (TAMs) from the M2 to the M1 phenotype [63]. This reprogramming promoted an immunologically active tumor microenvironment and inhibited tumor growth in mice, resulting in therapeutic effects [63].

Studies on CRISPR-Cas9 in gastric cancer have explored direct gene-editing approaches targeting major mutations, including TP53, CDKN2A, and KRAS, to restore tumor-suppressive function or disrupt oncogenic drivers [67,68,69], thereby recovering normal cellular functions. Large-scale CRISPR library screening can systematically identify genes essential for gastric cancer cell survival and contribute to therapeutic target discovery [64]. However, clinical application requires solutions to off-target editing and tumor-specific delivery. To address these challenges, several strategies to optimize delivery, including modulation of nanocarrier size, are currently being investigated [62].

Current research on CRISPR/Cas-based cancer therapy has largely focused on two complementary approaches: direct editing of tumor-associated genes and engineering of immune cells [15]. For example, in cervical cancer models driven by human papillomavirus (HPV) infection, CRISPR/Cas9-mediated disruption of the viral E6 and E7 oncogenes restored the activity of the tumor suppressors p53 and retinoblastoma protein (Rb), leading to apoptosis and tumor regression [15]. Systemic delivery of the CRISPR/Cas9 components using PEGylated liposomes resulted in efficient tumor elimination and prolonged survival in preclinical models [15]. In parallel, CRISPR/Cas9-mediated disruption of PDCD1 (encoding PD-1) in T cells enhanced cytotoxic activity and demonstrated safety and feasibility in early clinical trials [16,65]. However, subsequent studies revealed that tumor cell-intrinsic PD-1 may function as a tumor suppressor in certain cancer types, highlighting the context-dependent biological roles of PD-1 [65,66]. Overall, these results indicate that CRISPR/Cas-mediated cancer treatment is progressing on two fronts—tumor gene editing and immune cell engineering—and the translation to the clinic in the future will depend on enhancing delivery efficiency, reducing off-target effects, and gaining an accurate comprehension of tumor–immune interactions [15,66].

The development of cancer is driven by activating mutations in proto-oncogenes and loss-of-function mutations in tumor suppressor genes, which together disrupt the normal regulation of cell proliferation and survival [70]. These genetic alterations have also become important targets for CRISPR-Cas9, which has been applied both to validate candidate therapeutic targets and to engineer anti-tumor immune responses [71,72]. For example, CRISPR/Cas9-mediated knockout of WHSC1, a transcriptional target of the oncogene HMGA2, suppresses colon cancer cell proliferation and enhances chemosensitivity [71]. A more advanced application is adoptive T-cell therapy, in which CRISPR/Cas9 is used to disrupt the endogenous T-cell receptor (TCR) genes (TRAC and TRBC) and the immune checkpoint gene PDCD1 (encoding PD-1), followed by introduction of an NY-ESO-1-specific TCR transgene [73]. In the first-in-human clinical trial, the edited T cells successfully engrafted, persisted for several months, and trafficked to tumor sites. This study confirmed the feasibility and safety of multiplex CRISPR editing, but also identified locus-dependent variation in editing efficiency and rare chromosomal translocations. These limitations warrant further optimization and long-term clinical evaluation [73]. A similar strategy has also been adopted for CAR T-cell engineering by disrupting inhibitory immune checkpoint genes and inserting the CAR transgene into the TRAC locus. This approach eliminates endogenous T-cell receptor (TCR) expression, while promoting more uniform CAR expression [72]. In addition, this strategy is being extended to the development of “off-the-shelf” universal CAR-T products using healthy donor-derived or iPSC-derived T cells [72].

The translational relevance of chromosomal rearrangements was highlighted in a first-in-human trial of multiplex CRISPR-Cas9-edited T cells [71]. Simultaneous editing of *TRAC*, *TRBC*, and *PDCD1* resulted in chromosomal translocations in all manufactured T-cell products [71]. These rearrangements declined in frequency over time in vivo, with no evidence that they conferred a growth advantage to the edited cells. At the last time points assessed for each patient (days 30, 150, and 170), most translocations were at the limit of detection or undetectable, whereas the 9.3-kb *TRBC1:TRBC2* deletion remained detectable in all three patients [71]. These observations illustrate that the simultaneous induction of multiple DNA double-strand breaks during multiplex editing can generate chromosomal rearrangements that represent a genotoxicity concern independent of on-target editing efficiency.

Yin et al. subsequently showed that a substantial proportion of Cas9-induced translocations arises from repeated cleavage of target sites that undergo perfect rejoining, in which the original target sequence is restored and remains susceptible to Cas9 cleavage [74]. High-fidelity SpCas9 variants did not effectively suppress this mechanism [74]. To address this limitation, the authors fused SpCas9 with an optimized TREX2 exonuclease to generate Cas9TX, which promotes end processing and reduces perfect rejoining and subsequent repeated cleavage. Cas9TX nearly eliminated chromosomal translocations among *TRAC*, *TRBC*, and *PDCD1* during multiplex editing of CAR T cells while maintaining editing efficiency and antitumor cytotoxic activity [74].

Taken together, these findings indicate that evaluation of multiplex CRISPR-Cas9 editing strategies should extend beyond on-target efficiency to include structural genomic alterations such as chromosomal translocations. Engineered nuclease fusions designed to suppress repeated cleavage—rather than sequence-specificity variants alone—represent a promising strategy for improving the genomic safety of multiplex gene-editing therapeutics.

### 7.2. Advanced Delivery Platforms for CRISPR Therapeutics

The clinical translation of CRISPR-Cas9 therapeutics continues to be limited by the lack of efficient and safe delivery systems [13]. Tan et al. introduced dual-responsive dendrimer-based polymer nanocarriers (denoting a generation-5 PAMAM dendrimer dual-functionalized with phenylboronate and lipoic acid (GBLA)series) modified with phenylboronic acid and lipoic acid, which enhance Cas9 ribonucleoprotein (RNP) delivery by improving protein loading, promoting endosomal escape, and facilitating cytosolic release [13]. Notably, GBLA-22 successfully delivered Cas9 RNP targeting NLRP3 inflammasomes to reduce inflammation in a psoriasis mouse model, demonstrating the therapeutic potential of non-viral delivery approaches [13].

Beyond polymer-based nanocarriers, exosomes have also been explored as a non-viral delivery platform. One such system loaded Cas9 RNPs into hepatic stellate cell-derived exosomes via electroporation [75]. This approach enabled efficient cytosolic delivery and preferential accumulation in the liver, leading to targeted editing of PUMA, CcnE1, and KAT5 to ameliorate acute liver injury, chronic fibrosis, and hepatocellular carcinoma in mouse models [75]. This study illustrates that exosomes provide natural biocompatibility and tissue-specific tropism, positioning them as promising non-viral vectors.

A cell-based CRISPR delivery strategy using cryo-shocked A549 tumor cells has recently been developed [14]. Liquid nitrogen treatment abolished their tumorigenicity while preserving their surface architecture and membrane receptor expression [14]. The preserved CD44 receptors enabled the attachment of lipofectamine-encapsulated CRISPR/Cas9 plasmids targeting CDK4, generating the LNTLipopCas9/gCDK4 delivery system. Following systemic administration, this platform preferentially accumulated in the lungs, achieving approximately fourfold higher drug accumulation than conventional lipofectamine delivery and efficiently targeting KRAS-mutant non-small cell lung cancer (NSCLC) [14]. CRISPR/Cas9-mediated disruption of CDK4 induced synthetic lethality, which triggered marked tumor regression and significantly prolonged survival in mouse models [14].

Viral vectors remain another major delivery platform, though AAV in particular faces an intrinsic packaging limitation, since its ~4.5 kb capacity leaves little room to accommodate SpCas9 (~4.2 kb) together with its sgRNA cassette in a single vector. To address this, a smaller ortholog, SaCas9—over 1 kb shorter than SpCas9—was packaged with a U6-driven sgRNA and a TBG promoter-driven Cas9 cassette into a single AAV2/8 vector and delivered intravenously to target *Pcsk9* in the mouse liver [76]. This achieved over 40% indel formation, a ~95% reduction in serum Pcsk9, and a ~40% reduction in total cholesterol [76]. This single-vector strategy sidesteps the dual-vector workarounds other groups have needed for full-length SpCas9, though the size problem is only pushed back, not solved—little room remains for larger tools like base or prime editors. Whether prolonged Cas9 expression from the AAV episome carries added immunogenic risk is a question this study, conducted over a four-week window in mouse liver, does not directly address.

The blood–brain barrier (BBB) is a major obstacle to the clinical translation of CRISPR/Cas9-derived systems including dCas9-based regulators and prime editors. Approximately 98% of neurotherapeutic agents fail to cross the barrier systemically, and macromolecules are blocked almost entirely. As a result, large Cas9-derived complexes cannot passively reach neuronal targets, making BBB penetration a primary bottleneck for CNS-directed gene therapy [77]. Several strategies have been explored to overcome this limitation. Among them, AAV9 is notable for its intrinsic ability to cross the BBB, although its restricted packaging capacity and immunogenicity remain significant concerns. Engineered nanoparticles such as lipid nanoparticles (LNPs) require additional targeting modifications, since conventional formulations are preferentially distributed to peripheral organs such as the liver rather than the brain. Ligand-mediated receptor targeting and non-invasive routes, including intranasal administration, have been investigated as alternative approaches to bypass the barrier [77]. However, efficient and broadly distributed delivery of Cas9-derived systems to the brain remains unresolved [77]. Overall, BBB penetration is recognized as a central requirement for the successful clinical translation of CRISPR/Cas9-derived therapies in neurological disease. Continued progress will depend on the development of delivery platforms that balance efficiency, specificity and long-term safety [77].

However, delivery efficiency alone does not guarantee clinical success, as immune responses to the Cas9 protein represent an additional consideration [78]. Because *Staphylococcus aureus* Cas9 (SaCas9) and SpCas9 are derived from bacteria that commonly infect humans, preexisting humoral and cellular immunity against these orthologs has been detected in healthy individuals [79]. Consistent with the potential immunogenicity of Cas9, anti-Cas9 antibodies developed in all treated patients in a phase 1 in vivo clinical trial targeting *KLKB1*, although no discernible effects on pharmacokinetics, pharmacodynamics, or safety were observed following a single dose [78]. These findings highlight the importance of evaluating Cas9 immunogenicity, particularly when developing delivery strategies intended for repeated administration [78,79].

### 7.3. Infectious Disease Therapy

In infectious diseases, CRISPR/Cas technology has been explored for direct pathogen targeting, host-gene editing to inhibit infection, and the development of highly sensitive diagnostic platforms [80]. A representative direct pathogen-targeting strategy involves engineering bacteriophages to deliver CRISPR/Cas systems that selectively eliminate pathogenic bacteria [80]. To broaden bacterial host range, multiple phage candidates were combined and engineered to recognize diverse bacterial receptors. Based on this approach, SNIPR001 was developed as a phage cocktail carrying a Type I-E CRISPR-Cas system that targets multiple essential bacterial genomic sequences. This design reduces the likelihood of resistance while maintaining long-term stability [80]. In animal models, SNIPR001 significantly reduced the burden of target *E. coli* strains [80]. It also exhibited potent bactericidal activity against multidrug-resistant *E. coli* while sparing the surrounding gut microbiota, and its safety was demonstrated following oral administration in minipigs [80].

Editing host genes involved in viral entry offers a potential strategy for preventing viral infection. CRISPR/Cas9 has been investigated to disrupt host factors required for viral entry, such as the HIV co-receptor CCR5 [81]. CRISPR/Cas systems have also been used to directly target and eliminate viral genomes following infection [81]. Gene correction has also been demonstrated in patient-derived induced pluripotent stem cells, pointing to possible use beyond viral disease [81]. Clinical translation, however, is still constrained by delivery efficiency and editing specificity in vivo. Immune reactions to edited cells and the broader ethical questions raised by permanent genetic modification add further layers of difficulty.

### 7.4. Neurological Disorder Therapy

Neurodegenerative diseases present with diverse clinical manifestations, yet they converge on several common molecular abnormalities. Genetic mutations, risk variants, and repeat expansions can promote the misfolding and aggregation of disease-associated proteins, disrupting cellular homeostasis and contributing to neuronal dysfunction and degeneration. In Alzheimer’s disease, pathogenic mutations in amyloid precursor protein (*APP*) promote amyloid-β (Aβ) accumulation, whereas the apolipoprotein E (*APOE*) ε4 allele is a major genetic risk factor that influences Aβ metabolism and other disease-associated pathways [82,83]. Expanded CAG repeats in the huntingtin gene (*HTT*) result in the production of toxic mutant huntingtin protein [84]. Increased *SNCA* expression promotes α-synuclein accumulation, while GGC repeat expansions in *NOTCH2NLC* result in the production and accumulation of toxic polyglycine (polyG) proteins [85,86]. Most existing treatments primarily alleviate symptoms or modify specific disease processes without correcting the underlying genetic abnormalities, which has driven interest in CRISPR-based therapeutic approaches. Collectively, these disorders illustrate how CRISPR-based strategies could target disease-associated genetic alterations and downstream disruptions in protein homeostasis [81].

#### 7.4.1. Alzheimer’s Disease

**Figure 4:**
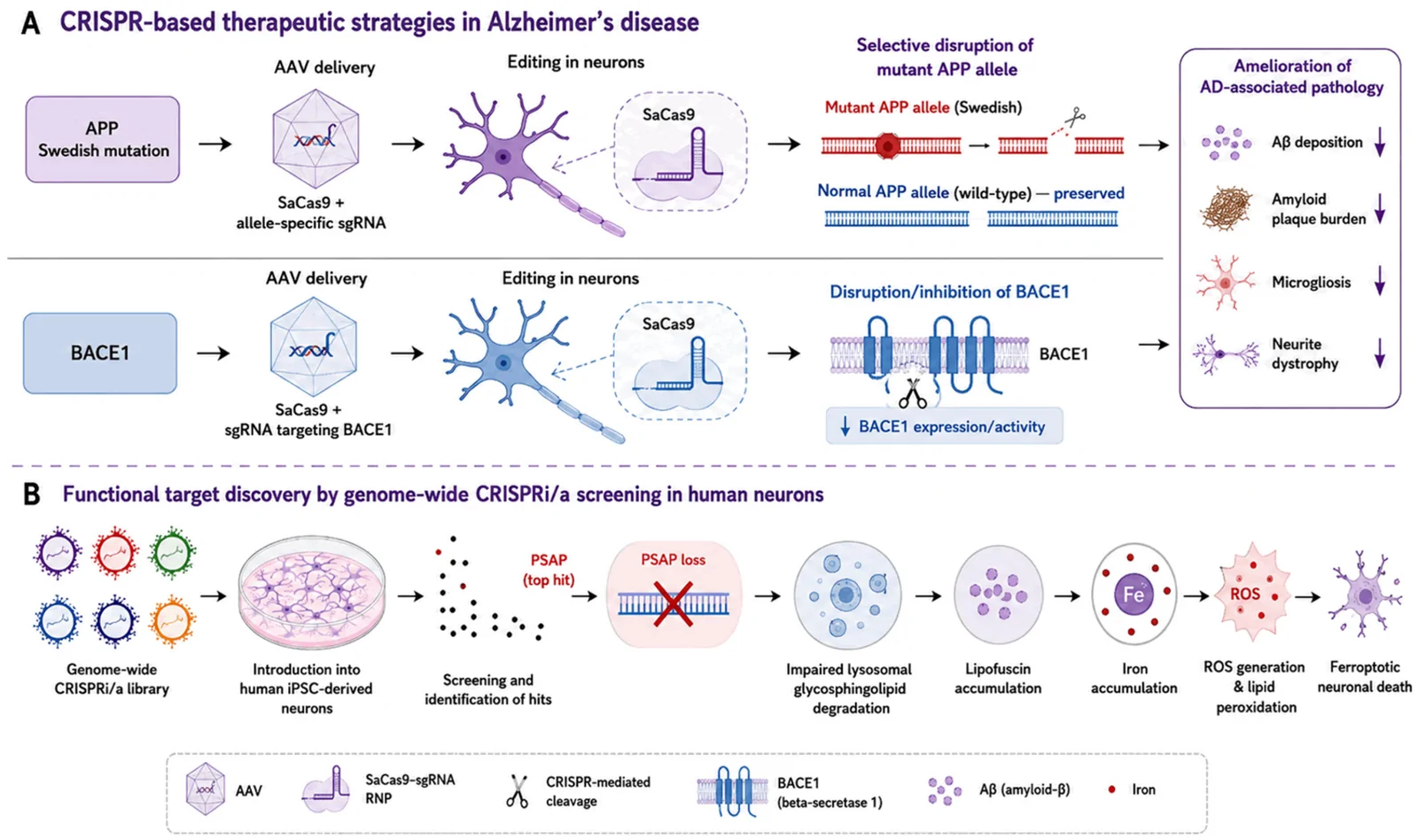
CRISPR-based therapeutic strategies and functional target discovery relevant to Alzheimer’s disease. (**A**) CRISPR-based therapeutic strategies for Alzheimer’s disease. SaCas9, delivered with an allele-specific sgRNA via AAV, selectively disrupts the mutant Swedish *APP* allele while preserving the wild-type allele, thereby reducing amyloid-β (Aβ) deposition, amyloid plaque burden, microgliosis, and neurite dystrophy. CRISPR-based modulation of other Alzheimer’s disease-associated genes, including *APOE* and *BACE1*, provides additional strategies for investigating and potentially modifying disease-associated pathways. (**B**) Functional target discovery by genome-wide CRISPRi/a screening. A genome-wide CRISPRi/a screen in human iPSC-derived neurons identified prosaposin (*PSAP*) as a key regulator of neuronal vulnerability. Loss of *PSAP* impairs lysosomal glycosphingolipid degradation, leading to lipofuscin accumulation, iron dysregulation, increased oxidative stress and lipid peroxidation, and ultimately ferroptotic neuronal death, linking lysosomal dysfunction to mechanisms of neurodegeneration.

In Alzheimer’s disease, allele-specific editing and pathway modulation represent promising therapeutic strategies. The Swedish mutation in the amyloid precursor protein (*APP*) gene was selectively disrupted using *Staphylococcus aureus* Cas9 (SaCas9) delivered by adeno-associated virus (AAV) vectors, resulting in reduced amyloid-β (Aβ) deposition, amyloid plaque burden, microgliosis, and neurite dystrophy [82,87]. Additional CRISPR-based approaches have targeted AD-associated genes and pathways, including *APOE* and β-site amyloid precursor protein cleaving enzyme 1 (*BACE1*), to investigate and potentially ameliorate disease-associated lipid and amyloid dysregulation (Figure 4A) [83,88]. Genome-wide CRISPRi/a screening in human iPSC-derived neurons further identified prosaposin (*PSAP*) as a key regulator of neuronal vulnerability. Loss of *PSAP* impaired lysosomal glycosphingolipid degradation, leading to lipofuscin accumulation, iron dyshomeostasis, lipid peroxidation, and ultimately ferroptotic neuronal death, thereby linking lysosomal dysfunction to mechanisms of neurodegeneration (Figure 4B) [89].

#### 7.4.2. Huntington’s Disease

**Figure 5:**
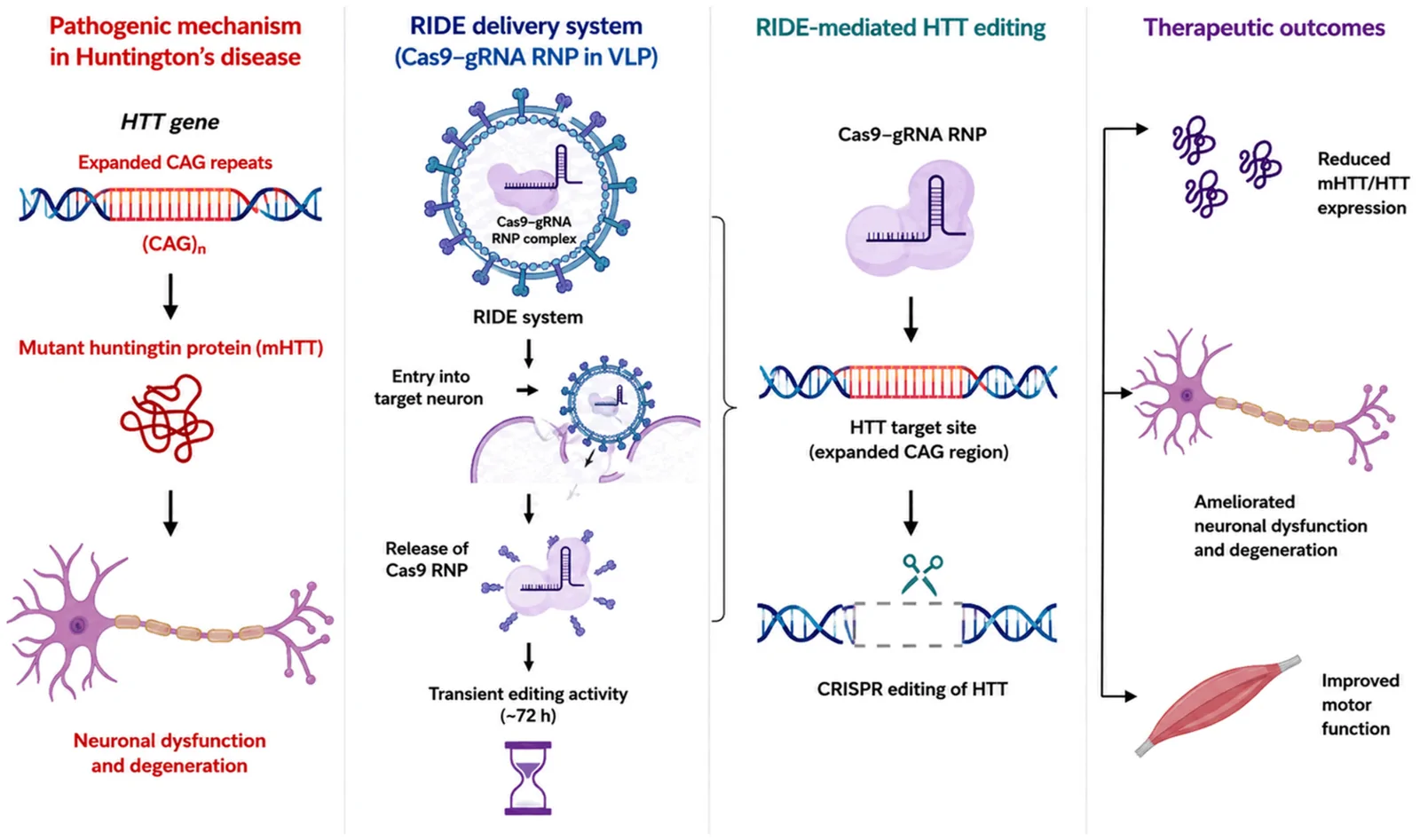
RIDE-mediated transient CRISPR-Cas9 delivery for Huntington’s disease. Expanded CAG repeats in *HTT* lead to the production and accumulation of mutant huntingtin (mHTT), resulting in neuronal dysfunction and degeneration. The RNP Integrating with Designer Envelope (RIDE) system packages preassembled Cas9–guide RNA ribonucleoprotein (RNP) complexes into virus-like particles (VLPs) for targeted delivery to neurons. Following cellular entry, Cas9 RNPs are released and transiently edit *HTT*, thereby avoiding prolonged nuclease exposure. RIDE-mediated *HTT* editing reduces mHTT levels and ameliorates disease-associated phenotypes, including motor deficits, in preclinical models of Huntington’s disease.

Huntington’s disease is caused by expanded CAG repeats in *HTT*, resulting in the production and accumulation of mutant huntingtin (mHTT) protein [84]. RNP Integrating with Designer Envelope (RIDE) is a customizable virus-like particle (VLP)-based delivery platform that delivers CRISPR-Cas9 as a preassembled ribonucleoprotein (RNP) complex, enabling transient genome editing [90]. Following RIDE-mediated delivery, Cas9 protein became barely detectable within approximately 72 h, thereby limiting prolonged nuclease exposure. In preclinical models of Huntington’s disease, RIDE-mediated *HTT* editing reduced HTT expression and ameliorated motor deficits (Figure 5) [84,90]. Other CRISPR-based strategies have employed non-allele-specific or allele-selective targeting of *HTT* to reduce mHTT expression while minimizing disruption of normal huntingtin function (Figure 5) [91]. Together, these findings highlight transient RNP delivery and selective *HTT* targeting as promising strategies for CRISPR-based treatment of Huntington’s disease.

#### 7.4.3. Neuronal Intranuclear Inclusion Disease (NIID)

Neuronal Intranuclear Inclusion Disease (NIID) shares mechanistic similarity with Huntington’s disease, as both are driven by repeat expansions. Specifically, Huntington’s disease involves expanded CAG repeats in the HTT gene leading to toxic huntingtin protein accumulation [84,90], whereas NIID is caused by GGC repeat expansion in the *NOTCH2NLC* gene, resulting in polyG protein aggregation [86]. A CRISPR/Cas9 dual-sgRNA strategy combined with homology-directed repair (HDR) successfully excised the expanded GGC repeats, reducing polyG protein accumulation and ameliorating molecular, neuropathological, and behavioral deficits [86]. Systemic delivery using AAV-PHP.eB (engineered capsid variant) enabled widespread central nervous system transduction and improved neuronal survival [86]. By targeting the repeat expansion itself rather than downstream effects, this approach directly addressed the underlying genetic cause of NIID. Furthermore, antisense oligonucleotide (ASO) therapy complemented CRISPR editing by targeting repeat-associated RNA toxicity [92]. ASOs rescued ribosomal RNA homeostasis, stabilized chromatin architecture, and mitigated cellular senescence [92]. This dual approach, combining genomic excision of pathogenic repeats with transcriptomic modulation, provides proof-of-concept for synergistic CRISPR and ASO strategies that may help restore cellular homeostasis and prevent progressive neurodegeneration in NIID.

Recent CRISPR-based studies in Parkinson’s disease have focused on epigenome editing to regulate disease-associated gene expression. Fusion of dCas9 with DNA methyltransferase 3A (DNMT3A) enabled targeted methylation of CpG islands within SNCA intron 1, increasing DNA methylation and reducing α-synuclein expression by approximately 30% [93]. This intervention improved mitochondrial function and enhanced neuronal survival under oxidative stress [93]. Complementary CRISPRi strategies targeting SNCA regulatory elements reduced α-synuclein aggregation in dopaminergic neurons, thereby improving neuronal viability and motor function. This indicates that epigenome editing is a potential treatment approach for Parkinson’s disease [85].

### 7.5. Other Emerging Therapeutic Applications

In the RIDE system described earlier in Huntington’s disease, its transient delivery mode shortens Cas9 expression, which helps limit off-target effects, reduce immune reactions against Cas proteins, and lower cytotoxicity [90]. As a result, RIDE shows strong potential for retinal gene therapy and other in vivo applications [90]. Surface modification of the VLP allows targeting of specific retinal cell populations, and this improves editing selectivity and safety [90].

In preclinical retinal models, vascular endothelial growth factor A (VEGF-A)-targeted genome editing markedly reduced pathological neovascularization and maintained retinal structure and visual function [90]. These outcomes highlight its therapeutic efficacy and safety in retinal applications [90]. Before clinical translation can be achieved, important hurdles must be addressed. These include optimizing intraocular distribution and penetration across retinal layers, improving off-target detection sensitivity, and evaluating long-term immunogenicity. In addition, scalable GMP-compatible manufacturing processes and stable formulations for VLP-mediated RNP delivery are required [90].

Sickle cell disease (SCD) is caused by a single-point mutation in the β-globin (HBB) gene, resulting in the production of hemoglobin S (HbS), which polymerizes under deoxygenated conditions and causes red blood cell sickling. Ex vivo genome editing of autologous hematopoietic stem and progenitor cells (HSPCs) using high-fidelity Cas9 ribonucleoproteins (RNPs) in combination with a recombinant adeno-associated virus serotype 6 (rAAV6) donor template achieved up to 60% allelic correction at a clinically relevant scale, restored normal hemoglobin expression, and maintained approximately 20% corrected alleles following multilineage engraftment in NOD-scid IL2Rγnull (NSG) mice [17]. In long-term safety studies, no abnormal hematopoiesis, genotoxicity, or tumorigenicity was observed. Thus, these findings support gene-corrected autologous hematopoietic stem cell transplantation as a durable, donor-independent therapeutic option for sickle cell disease (SCD) [17].

The first successful clinical translation study to treat SCD was performed by Sharma et al. [94], in which a tiling CRISPR-Cas9 screen tested 72 different guide RNAs targeting the HBG1/HBG2 promoter region in CD34+ cells from healthy donors. This screen identified the guide (gRNA-68) that produced the highest proportion of fetal hemoglobin-positive erythroblasts. In preclinical work, gRNA-68-edited CD34+ cells showed durable on-target editing with no detected off-target mutations. This study provides early clinical proof-of-concept that direct promoter disruption of HBG1/HBG2 can durably reactivate fetal hemoglobin production and may offer a viable CRISPR-based therapeutic strategy for severe sickle cell disease [94].

CRISPR technology is now used in genetic disease research and regenerative medicine, including disease modeling, gene correction, xenotransplantation, and biopharmaceutical production. CRISPR can correct disease-causing mutations in patient-derived iPSCs, while retaining their patient-specific genetic background. These corrected cells can be used for disease modeling, drug discovery, and the development of personalized regenerative therapies [81,95]. Multiplex genome editing has also been used to generate pigs lacking genes associated with transplant rejection and porcine endogenous retrovirus (PERV) transmission [96]. Genetically engineered pigs have additionally been used to express human therapeutic proteins, indicating another possible application in biopharmaceutical production [96]. Further development requires improved editing specificity and tissue-selective delivery, together with better control of immune responses. The long-term genomic stability and safety of edited cells and tissues must also be evaluated before broader clinical application.

### 7.6. Safety and Genomic Integrity of CRISPR Therapeutics

CRISPR-Cas9 safety concerns extend beyond conventional off-target effects. Kosicki et al. showed that Cas9-induced double-strand breaks can produce large on-target deletions and complex genomic rearrangements that may be missed by short-range genotyping [97]. Leibowitz et al. further demonstrated that Cas9-mediated DNA breaks can generate micronuclei and chromosome bridges that initiate chromothripsis—a catastrophic, localized shattering and reassembly of chromosomes recognized in cancer genomes as a mechanism capable of generating tumor-suppressor loss and oncogenic rearrangements [98]. In addition, Jiang et al. found that CRISPR-induced DNA damage enriched for cells carrying mutations in a p53-associated network, including *TP53* itself and DNA damage-response genes such as *TP53BP1*, *CHEK2*, and *ATM*, raising concerns about the potential clonal expansion of cells with impaired DNA damage responses and tumor-suppressive functions [99]. Together, these findings indicate that safety assessments of therapeutic CRISPR should extend beyond conventional off-target analysis to include structural genomic alterations and potential selection of cells with DNA damage-response defects, underscoring the importance of long-term monitoring for potential genotoxic and oncogenic consequences [97,98,99].

### 7.7. Ethical and Regulatory Considerations in CRISPR Clinical Translation

Beyond technical performance, CRISPR-based therapeutics raise ethical, legal, and social challenges that a translational review should address directly. Heritable (germline) genome editing in particular could alter the human gene pool, since changes introduced into embryos, gametes, or germline cells are passed on to future generations—a consequence not shared by the somatic-cell therapies that make up the large majority of current clinical CRISPR applications. Ethical debate in this space centers on informed consent, equitable access, and the risk that genome-editing tools could be repurposed for non-therapeutic uses, such as germline enhancement, outside their intended medical context [100,101].

Global governance efforts have sought to keep pace with this rapidly advancing technology. The World Health Organization has called for genome editing to proceed only within clearly defined safety limits, with international cooperation and meaningful public engagement in decision-making [102]. At the national and regional level, the U.S. Food and Drug Administration (FDA) and the European Medicines Agency (EMA) have each established safety and risk-management frameworks specific to genome-editing technologies. A central challenge for regulators is balancing the need for appropriate oversight with the rapid pace of innovation. Although CRISPR-based medicines hold substantial therapeutic promise, premature clinical implementation without adequate safeguards could erode public trust in the technology. Therefore, CRISPR therapeutics should remain subject to coordinated oversight by relevant regulatory authorities to ensure consistent safety standards, promote equitable access where appropriate, and protect the public interest. Robust ethical and regulatory frameworks should be established before these technologies are adopted for widespread clinical use.

## 8. Diagnostic Development

### 8.1. General Overview of CRISPR-Based Diagnostics

CRISPR-based diagnostics take advantage of the specific gene-targeting ability of Cas enzymes and guide RNA (gRNA). This target-specific binding and cleavage activity underlies both genome engineering and diagnostic applications. CRISPR-Cas systems have been used to detect genetic variations such as single nucleotide polymorphisms (SNPs) [7], DNA methylation [103], disease-related genes [104], and microRNAs [103,105,106], and even proteins without target amplification [107]. These diagnostic platforms rely on sequence-specific target recognition, nuclease cleavage, and collateral cleavage activity, with readouts based on fluorescence, colorimetry, voltammetry, or electrochemical detection signals [8]. CRISPR-based diagnostics have expanded the options for rapid and sensitive detection of infectious diseases. In addition to Cas12- and Cas13-based platforms such as SHERLOCK [7] and DETECTR [108], Class 1 Cas3-based systems, including CONAN and REPLICA, can recognize diverse PAM sequences and detect repeat sequences or targets associated with viral and genetic diseases with single-nucleotide specificity [109]. Therefore, these platforms are promising for point-of-care detection and patient stratification.

### 8.2. Pathogen and Infectious Disease Diagnostics

#### 8.2.1. Detection of Bacterial Pathogens and Antibiotic Resistance

CRISPR/Cas technology is increasingly used to monitor antimicrobial-resistant bacteria and analyze resistance-associated pathogens. CRISPR/Cas12a-based assays facilitate the rapid identification of methicillin-resistant *Staphylococcus aureus* (MRSA) through the detection of the *mecA* gene, which confers methicillin resistance [110]. In another study, a CRISPR-mediated DNA fluorescence in situ hybridization (FISH) method was developed using a dCas9-sgRNA complex to specifically bind the mecA gene of MRSA [111]. While *mecA* remains the primary marker for MRSA detection, other resistance mechanisms such as mecC, efflux pumps, and altered penicillin-binding proteins (PBPs) should also be considered to strengthen clinical relevance. DNA isolated using nickel–nitrilotriacetic acid (Ni-NTA) magnetic beads and stained with SYBR Green I generated fluorescence signals exclusively in resistant strains, enabling rapid and sensitive detection without the need for culture-based methods [111].

CRISPR-based detection has also been applied to pathogens that are difficult to culture or identify by conventional methods, such as Mycoplasma pneumoniae and Mycobacterium tuberculosis. Traditional approaches for detecting *Mycoplasma pneumoniae* have often been slow and unreliable. The *Mycoplasma pneumoniae* recombinase polymerase amplification–CRISPR (MP-RPA-CRISPR) platform addressed these shortcomings by integrating RPA with Cas12b detection, operating at 37 °C [112]. Once activated, Cas12b triggered collateral cleavage of single-stranded DNA (ssDNA) probes, releasing fluorescence signals that could be detected in real time or visually under blue light. This portable system was highly sensitive and required no complex instrumentation, supporting its use in resource-limited clinical settings [112]. A multi-guide RNA assay for *Mycobacterium tuberculosis* (TB) selected gRNAs with enhanced trans-cleavage activity, which improved sensitivity and accelerated reaction velocity [113]. By preferentially exploiting trans-cleavage activity, the assay demonstrated the potential to simplify point-of-care tuberculosis (TB) testing without compromising diagnostic accuracy [113]. In another study done by Zhang et al., paired dCas9 proteins fused to luciferase fragments were designed to bind adjacent target sites in TB DNA [114]. When the target DNA was present, the fragments reconstituted luciferase activity, producing luminescence signals that enabled highly specific detection [114]. CRISPR-based approaches have also been extended to resistance genotyping, enabling detection of single-nucleotide variations and insertion–deletion (indel) mutations associated with multidrug-resistant tuberculosis [115].

#### 8.2.2. Multiplexed Pathogen Detection

**Figure 6:**
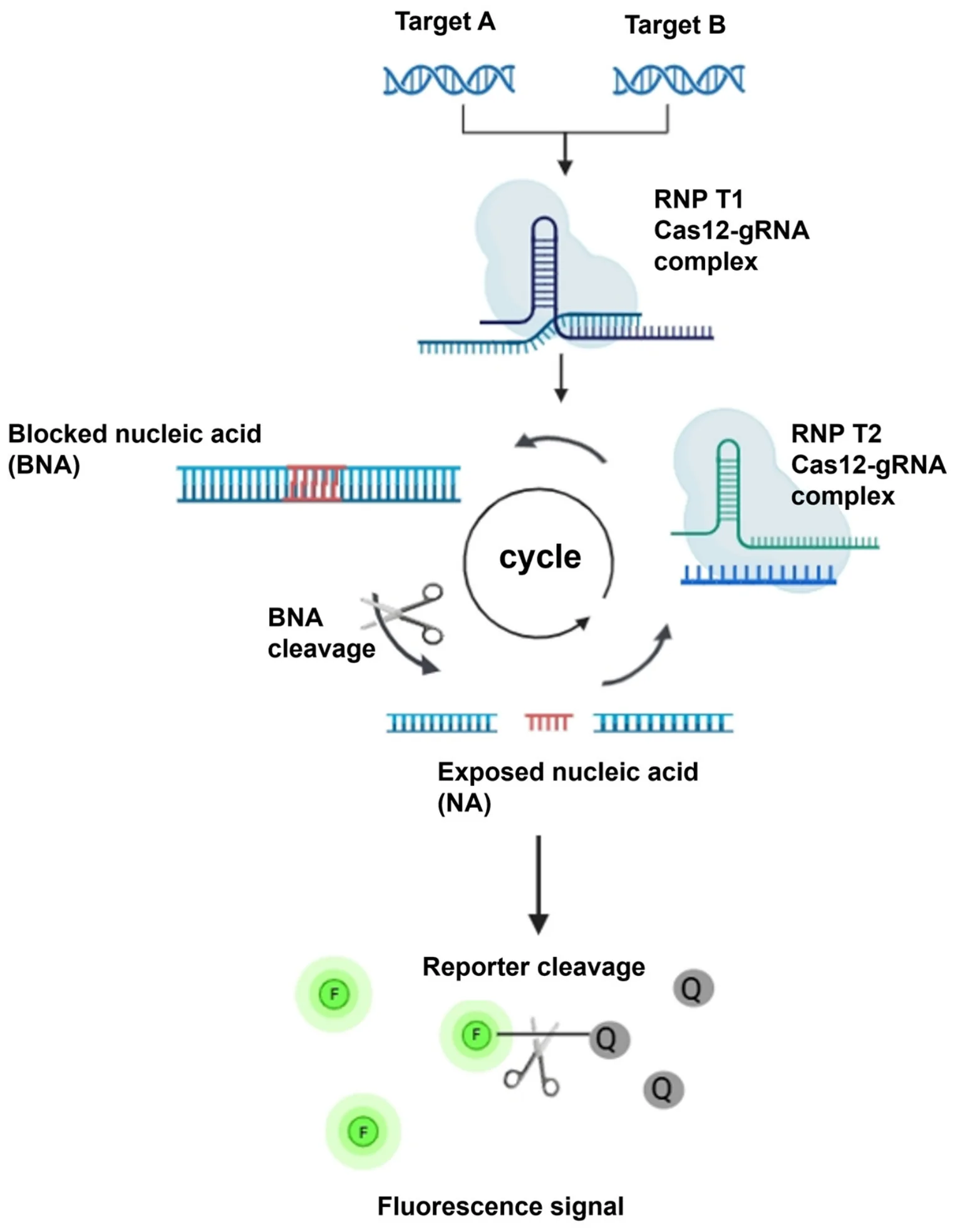
OR-gated CRISPR-Cascade assay for amplification-free pathogen detection. Recognition of either target A or target B by a Cas12–gRNA complex (RNP T1) triggers cleavage of blocked nucleic acids (BNAs), releasing nucleic acid activators that activate a second Cas12–gRNA complex (RNP T2). Subsequent BNA cleavage propagates a positive-feedback cascade, amplifying the detection signal without target preamplification. Activated Cas12 complexes then cleave quenched fluorescent reporters through collateral nuclease activity, generating a detectable fluorescence signal even from a single target molecule.

Recent CRISPR diagnostic platforms have moved toward multiplexed detection, allowing multiple pathogens or antimicrobial resistance markers to be identified simultaneously. Finding Low Abundance Sequences by Hybridization (FLASH) technology exploits Cas9-mediated sequence-specific cleavage to enrich target DNA before amplification, enabling the identification of antimicrobial resistance genes in *Staphylococcus* and *Plasmodium falciparum* [116]. Similarly, OR-gated CRISPR Cascade assays (Figure 6) couple target recognition to a self-amplifying CRISPR cascade. In this system, cleavage of blocked nucleic acids releases additional guide RNAs that amplify the detection signal, achieving attomolar (10^−18^ M) sensitivity without nucleic acid preamplification. The OR-gate design incorporates multiple guide RNAs. Any target sequence can initiate signal amplification, enabling rapid, amplification-free, multiplexed detection of MRSA, MSSA, E. coli, and HBV [117]. Combinatorial Arrayed Reactions for Multiplexed Evaluation of Nucleic acids (CARMEN) applies CRISPR-based detection to high-throughput, multiplexed panels capable of detecting multiple pathogens simultaneously [118]. Using microfluidic chips and optimized crRNA panels, CARMEN achieved detection limits as low as 10 copies/µL, outperforming reverse transcription quantitative polymerase chain reaction (RT-qPCR) in sensitivity [118]. Workflow time was reduced to four hours while maintaining accuracy across panels targeting viral hemorrhagic fevers, mosquito-borne viruses, and sexually transmitted infections. In clinical validation, CARMEN detected low-abundance infections that RT-qPCR missed [118].

#### 8.2.3. Rapid and Field-Deployable Viral Diagnostics

The CRISPR-assisted RNA recognition and detection (CARRD) platform senses RNA from pathogens such as human immunodeficiency virus (HIV) and hepatitis C virus (HCV) at room temperature, without the need for amplification [119]. CARRD detects RNA without target amplification and does not require temperature-controlled instrumentation [119]. Other CRISPR-based assays have been developed for respiratory viruses, including SARS-CoV-2. The DETECTR platform, built on Cas12a collateral cleavage activity, was adapted for SARS-CoV-2 detection [120]. DETECTR targets conserved regions of the viral genome and produces a fluorescent readout within approximately one hour [120]. Streamlined Highlighting of Infections to Navigate Epidemics (SHINE) combines Heating Unextracted Diagnostic Samples to Obliterate Nucleases (HUDSON)-based viral inactivation with single-step Cas13 detection. The assay analyzed saliva and nasopharyngeal swab samples without separate RNA extraction, produced results within 50 min, and showed 94% concordance with RT-qPCR [108]. SHERLOCK uses Cas13a collateral cleavage of reporter RNAs and achieves attomolar-level nucleic acid detection. The platform has been used to detect Zika and Dengue virus sequences, pathogenic bacteria, and cancer-associated mutations [7].

For African swine fever virus (ASFV), enzymatic recombinase amplification (ERA) was integrated with Cas12a to construct a multiplex assay targeting three viral genes (*E183L*, *K205R*, *C962R*) [121]. The combination of multiple primer–crRNA sets in a single reaction enhanced sensitivity and minimized false positives. Results visualized under blue light via a 6-carboxyfluorescein (FAM)–Black Hole Quencher (BHQ) reporter eliminated the need for specialized instrumentation, enabling on-site diagnosis [121]. A complementary platform coupled Cas12a trans-cleavage with urease-mediated signaling, providing both fluorescence and colorimetric readouts in an amplification-free, one-pot format [122]. For dengue virus, a one-pot RT-RPA/Cas12a assay targeting conserved genomic regions detected all four serotypes within 40 min [123]. The assay was compatible with lateral flow dipsticks, which minimized cross-contamination and simplified field deployment for outbreak surveillance [123]. For monkeypox virus (MPXV), a fiber-optic surface plasmon resonance (SPR) biosensor integrated with CRISPR/Cas12a detected the target down to 2.847 aM in buffer and 98.8 aM in simulated blood [124]. This miniature fiber-tip platform enabled rapid, label-free detection and genotyping directly from blood-derived specimens [124].

These platforms illustrate a common trajectory: CARRD detects viral RNA without amplification, ASFV assays multiplex three genes in a single reaction, dengue serotypes are resolved within 40 min, and MPXV biosensors reach 2.847 aM in buffer and 98.8 aM in simulated blood [119,121,122,123,124]. Despite differing targets and formats, each strategy reflects efforts to achieve shorter turnaround times and simpler workflows that may support use outside centralized laboratories.

### 8.3. Cancer Diagnostics Toward Non-Invasive and Early Detection

The application of CRISPR in oncology has expanded from functional studies of cancer genes to non-invasive cancer diagnostics. Early investigations established the causal roles of oncogenes and tumor suppressor genes in tumorigenesis by demonstrating that CRISPR-mediated gene disruption altered tumor growth and progression [125]. Subsequent studies focused on developing CRISPR-based platforms for sensitive detection of cancer biomarkers. For example, Chen et al. developed a CRISPR/Cas9-based assay for highly sensitive detection of circulating tumor DNA (ctDNA) [104]. The three-dimensional graphene-supported AuPtPd nanoflower (3D GR/AuPtPd) electrochemical biosensor combined with entropy-driven strand displacement reaction (ESDR) amplification detected ctDNA at femtomolar concentrations, suggesting potential for early cancer diagnosis [104]. To enhance ctDNA detection, a dual signal amplification strategy integrating hybridization chain reaction with proximity hybridization-regulated CRISPR/Cas12a was developed [126]. This method obtained a detection limit of 5.43 fM and was proven to work well in serum samples, indicating that it could be used for highly sensitive and non-invasive diagnostics. A CRISPR-based sensing strategy integrating aptamer-mediated capture of extracellular vesicles (EVs), T7 RNA polymerase amplification, and Cas13a detection was applied to identify EVs derived from lung cancer [127]. In clinical samples, the platform achieved ultrasensitive performance, producing significantly stronger signals in cancer patients than in healthy controls. These studies collectively demonstrate that CRISPR-based diagnostic platforms can detect multiple classes of circulating cancer biomarkers with high sensitivity. Additional validation in the clinic will be crucial for the adoption of these technologies for routine cancer diagnostics.

MicroRNAs (miRNAs) are dysregulated in cancer and remain stable in body fluids, making them attractive biomarkers for non-invasive diagnostics [128]. CRISPR-based assays detect oncogenic miRNAs using guide RNAs complementary to their target sequences [106]. Rolling circle amplification (RCA) coupled with dCas9 recognition amplified lung cancer-associated miRNAs into long DNA concatemers, generating robust fluorescent signals [129]. This approach significantly improved sensitivity compared to conventional quantitative polymerase chain reaction (qPCR), enabling detection of low-abundance miRNAs linked to lung cancer progression [129]. For point-of-care miRNA detection, Cas13a-based electrochemical biosensors were developed to detect medulloblastoma-associated miRNAs by coupling collateral cleavage activity to electrode readouts, producing measurable current changes without fluorescent dyes [106]. CRISPR-based cancer diagnostics have also expanded beyond nucleic acid targets. Cas12a-based aptamer sensors were engineered to bind hepatocellular carcinoma biomarker transforming growth factor beta 1 (TGF-β1), while collateral cleavage amplified the detection signal [107]. This system combined nucleic acid recognition with protein quantification, expanding CRISPR diagnostics beyond genetic targets [107]. Beyond genetic and protein biomarkers, CRISPR platforms have also enabled the sensitive detection of cancer-associated epigenetic alterations. Cas9-triggered CAS-EXPAR combines the sequence specificity of CRISPR/Cas9 with the rapid isothermal signal amplification of the Exponential Amplification Reaction (EXPAR) [103]. Once Cas9 cleaves its target, the released DNA fragments initiate EXPAR, generating exponential signal amplification for highly sensitive detection. When combined with bisulfite conversion, this strategy enabled precise quantification of DNA methylation, an important epigenetic biomarker for cancer classification, prognosis, and therapeutic decision-making [125]. These findings support the application of CRISPR-based diagnostics to non-invasive cancer screening [125].

### 8.4. Multiplexed Diagnostics

CRISPR-based diagnostic platforms have been developed for multiplexed detection of pathogens, genetic variants, and oncogenic biomarkers. Several nucleic acid detection platforms support multiplexed CRISPR diagnostics. As discussed earlier, Cas12- and Cas13-based systems such as SHERLOCK and DETECTR have demonstrated rapid nucleic acid detection with single-nucleotide resolution, and their collateral cleavage activity can be harnessed for multiplexed panels [7,8]. SHINE, described earlier, achieves extraction-free diagnostics within 50 min with high concordance to RT-qPCR [108]. OR-gated CRISPR cascades achieve single attomolar detection limits, while supporting multiplexed RNA target recognition [117]. They introduced a multiplexing OR-function logic gate for developing rapid and accurate diagnostics in clinical settings. Logical “OR” gating allows simultaneous detection of multiple pathogens, while maintaining rapid and accurate detection [117]. Ackerman et al. demonstrated massively multiplexed Cas13 assays capable of detecting over 4500 RNA targets simultaneously with attomolar sensitivity [130]. Another study by Arizti-Sanz et al. validated a streamlined Cas13-based diagnostic, achieving 90% sensitivity and 100% specificity in 50 nasopharyngeal patient samples with a total run time of 50 min [108]. Chow et al. reported that multiplexed CRISPR-Cas9 screening identified cooperative tumor suppressor losses, such as simultaneous tumor protein p53 (TP53), phosphatase and tensin homolog (PTEN) and neurofibromin 2 (NF2) deletions, which induced tumorigenic phenotypes in glioblastoma models [131]. These results underline the transformative potential of multiplexed CRISPR diagnostics in diseases with multiple possible etiologies or combinations of mutations at the origin of the disease in clinical practice [108,130,132].

The multiplexed gene-editing screens described earlier, which reveal which mutation combinations drive tumorigenesis, also inform the design of multiplexed therapeutic edits. In CRISPR-engineered T-cell therapy, T cells are simultaneously edited with Cas9 to remove the endogenous T-cell receptor (TCR) and the immune checkpoint molecule programmed cell death protein 1 (PD-1), while a cancer-specific TCR-encoding transgene is introduced [71]. This multiplex-edited product has demonstrated safety and disease stabilization in patients with refractory cancer [71]. This mirrors the logic of multiplexed diagnostic screening—coordinating edits across several genomic targets at once—but here the goal is treatment, not detection.

Another notable application of multiplexed CRISPR diagnostics is the integration of allele-specific recombinase polymerase amplification (AS-RPA) with CRISPR/Cas12a for genetic screening of common glucose-6-phosphate dehydrogenase (G6PD) mutations in Thailand [133]. This assay exploits mismatch discrimination and incorporates locked nucleic acids (LNAs) to sharpen target specificity, allowing reliable detection of clinically relevant variants. The platform delivers results within one hour while maintaining high sensitivity and specificity. The assay is also inexpensive and field-deployable, making it well-suited for endemic regions where G6PD deficiency poses a significant risk for patients receiving antimalarial therapy. These findings support the use of multiplexed CRISPR diagnostics for clinical decision-making in resource-limited settings [130,133].

## 9. Conclusions

Over the past decade, CRISPR-Cas technology has evolved from a bacterial adaptive immune mechanism into one of the most versatile platforms in biomedicine, spanning genome editing, epigenome reprogramming, transcriptional control, functional target discovery, and molecular diagnostics. The programmability and modularity of CRISPR/Cas effectors underlie its expansion along three interconnected directions. In biological discovery, CRISPR-based functional screening has been widely used to identify novel disease-associated genes and therapeutic targets across cancer and neurodegeneration. This approach provides a systematic route from genotype to mechanism. Building on these discoveries, engineered CRISPR platforms—from base editors and epigenome modifiers to multiplex-edited cell therapies—are advancing toward clinical application. Ongoing efforts to improve editing precision, delivery, and safety continue to narrow the gap between laboratory discovery and patient benefit. The collateral cleavage activity of Cas12 and Cas13 effectors has enabled a new generation of rapid, highly sensitive, and field-deployable detection platforms, such as SHERLOCK, DETECTR, and their derivatives. These platforms rival or complement PCR while extending testing capability to point-of-care and resource-limited settings. These three domains are not isolated from one another: therapeutic target discovery informs diagnostic biomarker selection, diagnostic platforms help validate therapeutic mechanisms, and shared Cas biochemistry underlies tools used across all three areas. This convergence reflects how closely basic discovery and clinical translation are now linked in CRISPR/Cas research.

Despite these advances, several limitations continue to constrain the clinical translation of CRISPR/Cas technology. Off-target effects and variable editing efficiency remain persistent concerns across genome, epigenome, and RNA-editing platforms, as illustrated by the guide-independent off-target activity observed even in specificity-optimized systems such as REPAIRv2. Delivery remains an equally significant bottleneck, as viral vectors face packaging and immunogenicity constraints while non-viral nanocarriers still require further optimization for in vivo efficiency and specificity. Clinical applications involving ex vivo or in vivo cell editing have demonstrated feasibility, but variability in editing outcomes and the risk of unintended genomic alterations point to the need for more rigorous safety profiling. In diagnostic development, translating CRISPR-based platforms into routine clinical use is similarly hindered by a lack of standardized protocols and harmonized regulatory frameworks.

Future advances in CRISPR/Cas technology are expected to be driven by several key developments. Technologically, deeper integration with microfluidics and artificial intelligence is expected to enable fully automated, sample-to-answer diagnostic systems. This integration is also expected to accelerate guide RNA design and off-target prediction. Beyond infectious disease detection, these advances are likely to extend applications into oncology, genetic disease screening, and other fields. Clinical implementation will require large-scale standardization, harmonized regulatory pathways, and continued collaboration across biology, engineering, and clinical medicine. Addressing these challenges will facilitate the translation of CRISPR-Cas diagnostics, target discovery, and therapeutics from laboratory research to routine clinical practice.

## Figures and Tables

**Table 1:** A comparative summary table of Cas9, Cas12, and Cas13 [3,4,8,19,20].

	Cas9	Cas12a	Cas13a/b
Target Substrate	Double-stranded DNA	Double-stranded DNA	Single-stranded RNA
PAM Requirements	NGG	TTTN (T-rich PAM)	-
Cleavage Properties	Blunt-end	Staggered-end	RNA cleavage with collateral RNA degradation
Editing Modalities	Knock-out, knock-in, base editing, prime editing	Knock-out, knock-in, limited base editing	RNA knockdown, RNA editing, diagnostics (SHERLOCK)
Specificity	Moderate	High specificity	High RNA target specificity
Advantages	Simple gRNA design	Flexible PAM recognition	RNA targeting capability
Limitations	Immunogenicity in vivo	Limited clinical validation, variable efficiency, potential immunogenicity	Collateral RNA cleavage undermines therapeutic safety, potential immunogenicity
Representative Applications	Gene therapy (sickle cell disease, ocular disorders)	Gene correction	Infectious disease diagnostics RNA-based disease research

## Data Availability

No new data were created or analyzed in this study. Data sharing is not applicable to this article.
